# Modulation of transient receptor potential (TRP) channels by plant derived substances used in over-the-counter cough and cold remedies

**DOI:** 10.1186/s12931-023-02347-z

**Published:** 2023-02-08

**Authors:** Rebecca J. Stinson, Alyn H. Morice, Laura R. Sadofsky

**Affiliations:** 1 Centre for Biomedicine, Hull York Medical School, The University of Hull, Cottingham Road, Hull, HU6 7RX UK; 2 Clinical Sciences Centre, Hull York Medical School, Castle Hill Hospital, Cottingham, Hull, HU16 5JQ UK

**Keywords:** Menthol, Camphor, Eucalyptus, Thymol, TRP channels, Cough, Cold, Over-the-counter therapies

## Abstract

**Background:**

Upper respiratory tract infections (URTIs) impact all age groups and have a significant economic and social burden on society, worldwide. Most URTIs are mild and self-limiting, but due to the wide range of possible causative agents, including Rhinovirus (hRV), Adenovirus, Respiratory Syncytial Virus (RSV), Coronavirus and Influenza, there is no single and effective treatment. Over-the-counter (OTC) remedies, including traditional medicines and those containing plant derived substances, help to alleviate symptoms including inflammation, pain, fever and cough.

**Purpose:**

This systematic review focuses on the role of the major plant derived substances in several OTC remedies used to treat cold symptoms, with a particular focus on the transient receptor potential (TRP) channels involved in pain and cough.

**Methods:**

Literature searches were done using Pubmed and Web of Science, with no date limitations, using the principles of the PRISMA statement. The search terms used were ‘TRP channel AND plant compound’, ‘cough AND plant compound’, ‘cough AND TRP channels AND plant compound’, ‘cough AND P2X3 AND plant compound’ and ‘P2X3 AND plant compound’ where plant compound represents menthol or camphor or eucalyptus or turpentine or thymol.

**Results:**

The literature reviewed showed that menthol activates TRPM8 and may inhibit respiratory reflexes reducing irritation and cough. Menthol has a bimodal action on TRPA1, but inhibition may have an analgesic effect. Eucalyptus also activates TRPM8 and inhibits TRPA1 whilst down regulating P2X3, aiding in the reduction of cough, pain and airway irritation. Camphor inhibits TRPA1 and the activation of TRPM8 may add to the effects of menthol. Activation of TRPV1 by camphor, may also have an analgesic effect.

**Conclusions:**

The literature suggests that these plant derived substances have multifaceted actions and can interact with the TRP ‘cough’ receptors. The plant derived substances used in cough and cold medicines have the potential to target multiple symptoms experienced during a cold.

## Introduction

Upper respiratory tract infections (URTIs) represent a significant global burden on society from both a social and economic perspective owing to high morbidity levels across all age groups [1]. The exact cost is difficult to estimate however, extrapolation from direct cost of healthcare, over-the-counter (OTC) cough and cold remedy sales, and loss of income suggest URTIs cost in excess of $20–40 billion annually in the United States (US) [2–5] and approximately £11 billion in the United Kingdom (UK) [6]. With a range of potential causative agents including human Rhinovirus (hRV), Adenovirus, Respiratory Syncytial Virus (RSV), Coronavirus and Influenza, URTIs cause a variety of symptoms [5]. Common symptoms include cough, nasal congestion and excessive mucus production [7]. Although the majority of URTIs are mild and self-limiting in nature [8], there is no single effective treatment for the troublesome symptoms [9]. The desire to alleviate symptoms has led to a number of OTC remedies, such as anti-inflammatories, analgesics and antipyretics to target fever and muscle pain, alongside H1 receptor antagonists, decongestants and nasal sprays which target nasal congestion. Furthermore, cough can be targeted through specific antitussive medicines [10–12]. A number of herbal and traditional remedies also exist to help alleviate symptoms including honey as an antitussive agent [13], saline solutions for nasal congestion and throat irritation [14], vitamins, mineral supplements and remedies purported to boost immune function [12, 15–17] and topical vapour rub ointments containing menthol, camphor and eucalyptus, which release therapeutic vapours aimed at reducing cough and congestion and making breathing easier [18].

Menthol, eucalyptus and camphor form the main ingredients in many herbal cough and cold remedies [19–21] and are the focus of this review. Whilst, historically these plants were chosen for their medicinal properties and the relief they provide from a number of symptoms when infused, steeped or heated to create herbal drinks, the investigation of the pharmacological mechanism of action is a more recent development. Scientific investigation into these mechanisms has been sparse and is still not fully understood [22]. Furthermore, each of these plant derived substances have a range of specific pharmacological activities that are potentially beneficial in the alleviation of cold symptoms and other diseases (Table 1).

**Table 1:** Medicinal properties and chemical structures of the plant derived substances frequently found in traditional herbal cough and cold remedies [22, 96, 97, 104, 114, 146–160]

	Menthol	Camphor	Eucalyptus oil	Turpentine oil	Thymol	Cedarleaf oil	Nutmeg oil
Plant	*Mentha x piperita* (Peppermint) and other members of the mint family	*Cinnamomum camphora* (Camphor Laurel)	*Eucalyptus globulus* (Tasmanian blue gum) and other members of the eucalyptus family	*Pinus Pinaster* (Maritime pine) and other members of the pine family	*Thymus vulgaris*	*Thuja orientalis* (Arbor vitae) and other members of the Cupressaceae family	*Myristica fragrans* (Fragrant nutmeg)
Chemical Structure of Main Pharmacologically Compound							
Medicinal properties described							
Antibacterial	✓	–	✓	–	–	✓	–
Analgesic	✓	✓	✓	–	–	–	✓
Anti-inflammatory	–	✓	✓	✓	–	–	✓
Antioxidant	–	–	✓	✓	–	✓	✓
Antiviral	–	–	–	✓	–	✓	–
Antimicrobial	–	–	✓	✓	✓	–	✓
Antitussive	✓	✓	–	–	✓	–	–
Antipyretic	–	–	–	–	✓	–	–
Expectorant	–	✓	–	✓	✓	–	–
Sedative	–	–	–	–	✓	–	–
Cooling effect	✓	–	–	–	–	–	–
Counter irritant	–	✓	–	–	–	–	–
Antipruritic	*	*	–	–	–	–	–
Antifungal	*	–	*	–	*	*	–
Antiseptic	*	–	–	*	–	–	*
Antispasmodic	–	*	–	–	*	–	–
H1 receptor antagonist	–	–	*	–	–	–	–
Antiparasitic	–	–	–	*	*	–	–
Astringent	–	–	–	–	–	*	–
Disinfectant	–	–	*	–	–	–	–

✓denotes medicinal properties most relevant to the management of cold symptoms

*denotes additional medicinal properties not specifically related to cold symptoms

There is encouraging evidence to suggest that transient receptor potential (TRP) cation channels may play a role in cough and airway inflammation. Furthermore, several of the plant derived substances used in traditional and herbal cough and cold remedies are known to modulate TRP channel function. In this systematic review we will explore how the most frequently found plant derived substances found in OTC cough and cold remedies potentially interact with TRP channels involved in the cough reflex creating the clinical effects observed.

## Role of TRP channels in cough

**Fig. 1:**
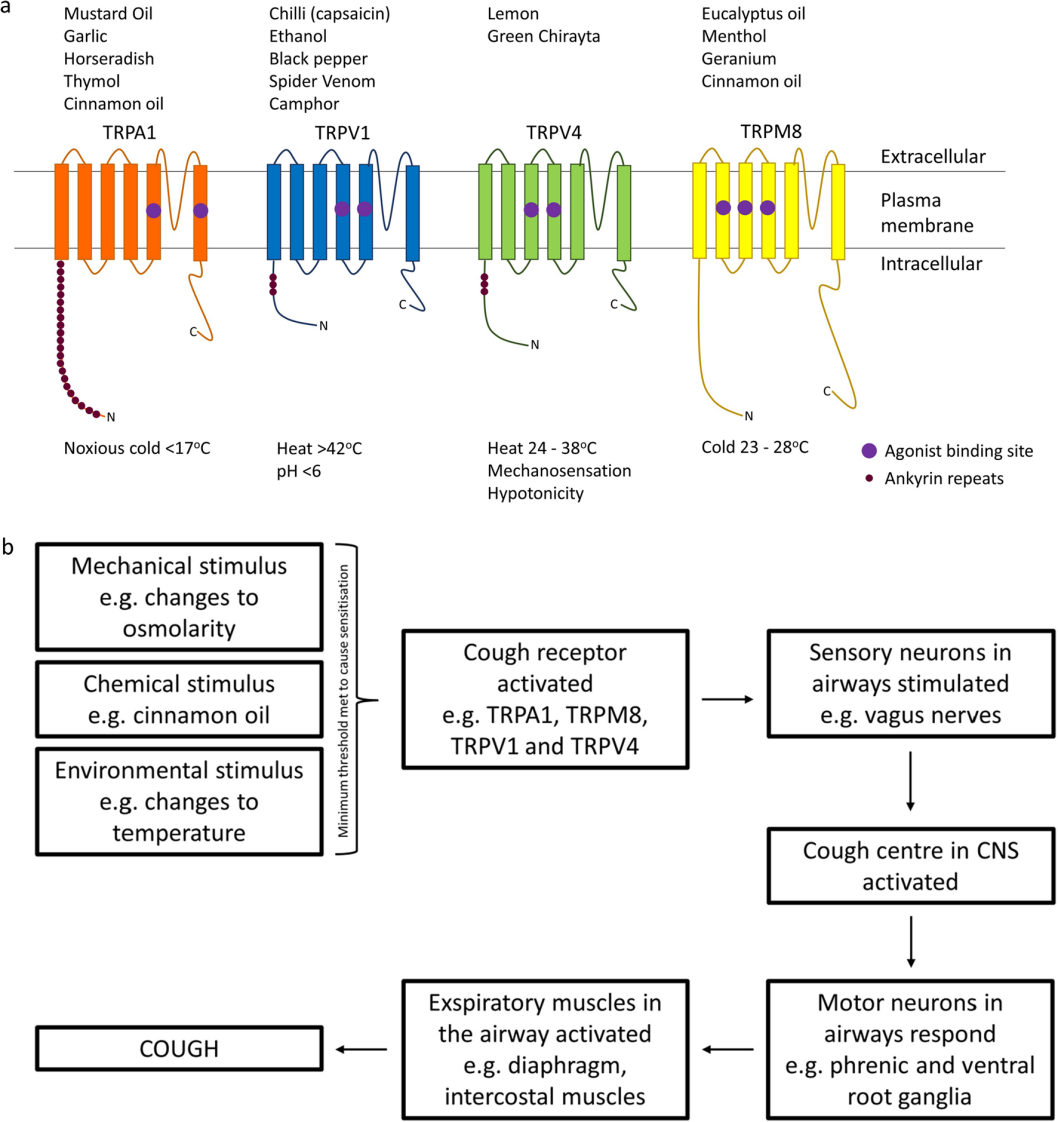
**a** Structural representation of the potential TRP channels involved in cough and airway hypersensitivity. Example chemicals which stimulate each TRP channel are shown above and the mechanical and physical stimuli below. **b** Flow diagram representing the steps involved in the cough reflex pathway in relation to the activation of TRP channels via relevant stimulants

Comprised of 28 members, mammalian TRP channels are a family of conserved transmembrane proteins, divided into six subfamilies; vanilloid (TRPV), melastatin (TRPM), mucolipin (TRPML), canonical (TRPC), ankyrin (TRPA) and polycystic (TRPP), each of which are further subdivided into individuals members [23]. TRP channels share a common structure, comprising of six transmembrane spanning proteins, assembled as a tetrameric channel. With differences between family members being derived from variations in the cytosolic N- and C-terminals [24]. Cough and airway hypersensitivity have been linked to upregulation of TRP channels on the sensory nerves in the respiratory tract [25], however, not all TRP channels are thought to function as cough receptors, those most noticeably involved include TRPV1, TRPV4, TRPM8 and TRPA1 (Fig. 1a). These receptors all play a role in airway sensation, responding to changes in temperature, pH, osmolarity, irritants and mechanical stretch [26] (Fig. 1b). Activation of these TRP channels occurs on reaching a threshold of tolerance to a stimulus, this opens the channels, enabling the movement of ions across the membrane and generation of an action potential, leading to the observed response [27, 28]. However, this activation can often be attenuated through the use of suitable antagonist compounds, which alter the channel response [29]. The threshold at which an action potential is propagated varies, with factors such as underlying respiratory disease and hyperstimulation having the potential to lead to hypersensitisation, desensitisation and down-regulation of TRP receptors [30]. For example, the TRPV1 role in cough is linked to the increased sensitivity to capsaicin seen in asthmatics and COPD patients, which suggests that during inflammation there is upregulation of TRPV1 expression and function [31].

### TRPA1

TRPA1, previously ANKTM1, is the only TRPA channel expressed in mammalian cells and acts as a non-selective cation channel. Found in fibroblasts, epithelial cells (including airway epithelia), melanocytes, smooth muscle cells and neurons [23, 32, 33]. TRPA1 is frequently co-localised on afferent neurons alongside TRPV1 however, activation of either receptor is dependent on the presence of specific stimuli [34]. TRPA1 is predominately activated by a wide range of chemicals including those with strong odours and tastes including garlic, horseradish, cinnamon oil, mustard oil and wasabi [23, 35]. Activation can occur via covalent modification of cysteine residues on the N-terminus of the receptor [34, 36]. In addition, TRPA1 is a thermoreceptor, being activated by noxious cold temperatures below 17 °C [37] and nociceptor, creating a potential target for pain relief and reducing bronchial hyperresponsiveness in asthmatics when exposed to inhaled irritants [38]. Furthermore, inhalation of TRPA1 agonists such as cinnamaldehyde are known to cause cough, confirming its role as a cough receptor [39].

### TRPM8

TRPM channels were first identified in tumour cells with expression linked to metastatic potential [23, 35]. TRPM8 is predominately expressed in neurons, but also taste papillae, testis, prostate, lungs [40], cornea [41], skin and bladder, weak expression is also observed in pulmonary smooth muscle and liver [42]. TRPM8 is activated by a number of chemical compounds, with the most extensively studied being menthol and eucalyptus [43, 44]. Additionally, TRPM8 is thermoregulated, being activated by cool temperatures between 23 and 28 °C [35] and shows evidence of responding to increases in osmolarity [41]. Furthermore, activation of TRPM8 by cooling compounds such as menthol, makes it a potential analgesic target, as activation can alleviate pain from inflammation and noxious heat [44]. Furthermore, activation could have an anti-inflammatory effect, whereby pro-inflammatory cytokine release is inhibited [32] which may limit activation of nerve fibres involved in cough, potentially providing an antitussive effect [45].

### TRPV1

TRPV1 is activated by vanilloid compounds most markedly that of capsaicin, in addition to camphor, black pepper, ethanol and garlic [35, 46–48]. Other chemical and physical stimuli include temperatures above 43 °C, low pH and spider toxin [24, 33, 35, 38]. TRPV1 is expressed in the liver, heart, pancreas and lungs [49] however, the most predominant expression is in afferent nerve fibres throughout the skin and gut, thus acting as both a thermoreceptor and nociceptor whereby it plays a key role in pain detection [23, 32, 33, 50]. The activation of afferent nerve fibres also plays a role in the airways, insofar as activation of TRPV1 by inhaled irritants results in increased mucus secretion, bronchoconstriction and an urge to cough. Furthermore, increased expression of TRPV1 is linked to chronic cough and hypersensitivity in chronic airway diseases [49, 51]. The activation of TRPV1 as well as TRPA1 can also occur via intracellular calcium, furthermore, the co-expression of the receptors can result in one channel sensitising the other [67]. Thus it is possible that during airway inflammation both receptors may be activated simultaneously [68], as such antitussive agents may be better targeted to both receptors rather than individual ones [34].

### TRPV4

TRPV4 is widely expressed throughout mammalian tissue including in the nervous system, heart, skin, kidneys, sweat and salivary glands, and lungs [52]. TRPV4 is activated by a number of mechanical stimuli including changes to osmolarity, mechanical stretch, shear stress and temperatures between 24 and 38 °C [33, 53]. Suggested TRPV4 functionality includes regulation of blood flow, ciliary action control, osmotic regulation, vasodilation and nociception [54]. The osmoregulation function of TRPV4 is of particular interest in relation to viral URTIs which can result in the upregulation of mucus and changes in viscosity, thus altering the hypotonicity of the mucus in airways, leading to the activation of TPRV4 [55, 56]. Furthermore, TRPV4 is implicated in respiratory function and disease, playing a role in endothelial and epithelial barrier integrity, smooth muscle constriction and regulation of inflammation, which if compromised can result in alveolar oedema [57, 58], whilst gene polymorphisms are linked to chronic obstructive pulmonary disease (COPD) [59]. TRPV4 has also been linked to the cough reflex owing to the production of adenosine triphosphate (ATP) in response to activation, which activates other receptors, namely purinergic receptor P2X3 [59].

### Involvement of P2X3 (TRPV4–ATP–P2X3 pathway)

**Fig. 2:**
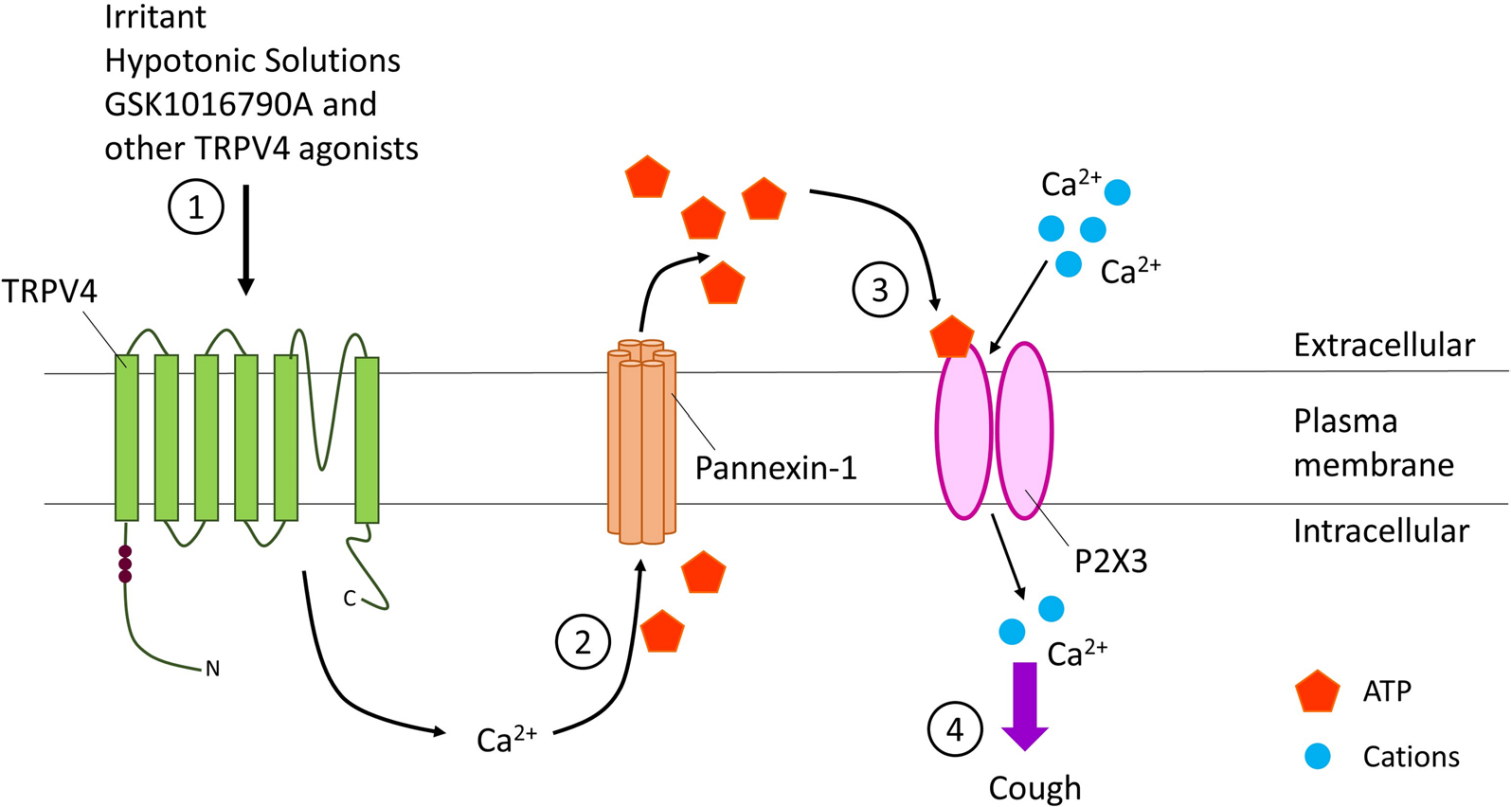
TRPV4-ATP-P2X3 pathway involved in the cough response. Proposed mechanism involves the activation of TRPV4 by hypotonic solutions, irritant or known agonist, leading to the influx of calcium ions into the cytosol (1). Activation of TRPV4 and the increase in intracellular calcium, leads to the release of ATP into the extracellular space mediated by pannexin-1 (2). Extracellular ATP activates P2X3 on sensory neurons creating an action potential in the sensory neurons of the airways (3) which may subsequently trigger the cough reflex (4)

As a common symptom of URTIs, cough is an area of significant interest as for some individuals acute cough can become chronic, lasting in excess of 8 weeks however, the mechanism involved has not been fully elucidated [55]. Of particular interest in this mechanism are the P2 purinergic receptors (P2R), specifically the P2XR, transmembrane cationic channels on sensory neurons, which are mediated by ATP [60]. The receptor of most interest is P2X3, whereby activation of TRPV4 causes the release of ATP, through pannexin-1, which subsequently activates P2X3 eliciting a cough response (Fig. 2) [59]. The cough response can be partially attenuated using a P2X3 antagonist, which shows promise for the treatment of chronic cough [61, 62] and thus may provide a potential target for the treatment of cough as a symptom of URTIs. Indeed, recently hRV-16 has been shown to increase ATP release by airway epithelial cells with and without secondary TRPV4 stimulation suggesting a role for ATP release in URTIs [63].

Of the aforementioned TRP channels, TRPA1 and TRPV1 have the most significant link to the cough mechanism, although TRPV4 has also been postulated to play some role being co-localised on the same sensory neurons and through the TRPV4-ATP-P2X3 pathway [59]. Interestingly, TRPA1 and TRPV1 antagonist have been shown to inhibit cough induced by irritants and agonists of the channels e.g. citric acid or capsaicin. However, therapies such as SB-705498 (TRPV1 antagonist) and GRC 17536 (TRPA1 antagonist) failed to reduce cough in chronic cough patients [64–66]. Importantly, the role of these TRP channel modulators in URTI associated cough have not yet been proven.

## Systematic review

**Fig. 3:**
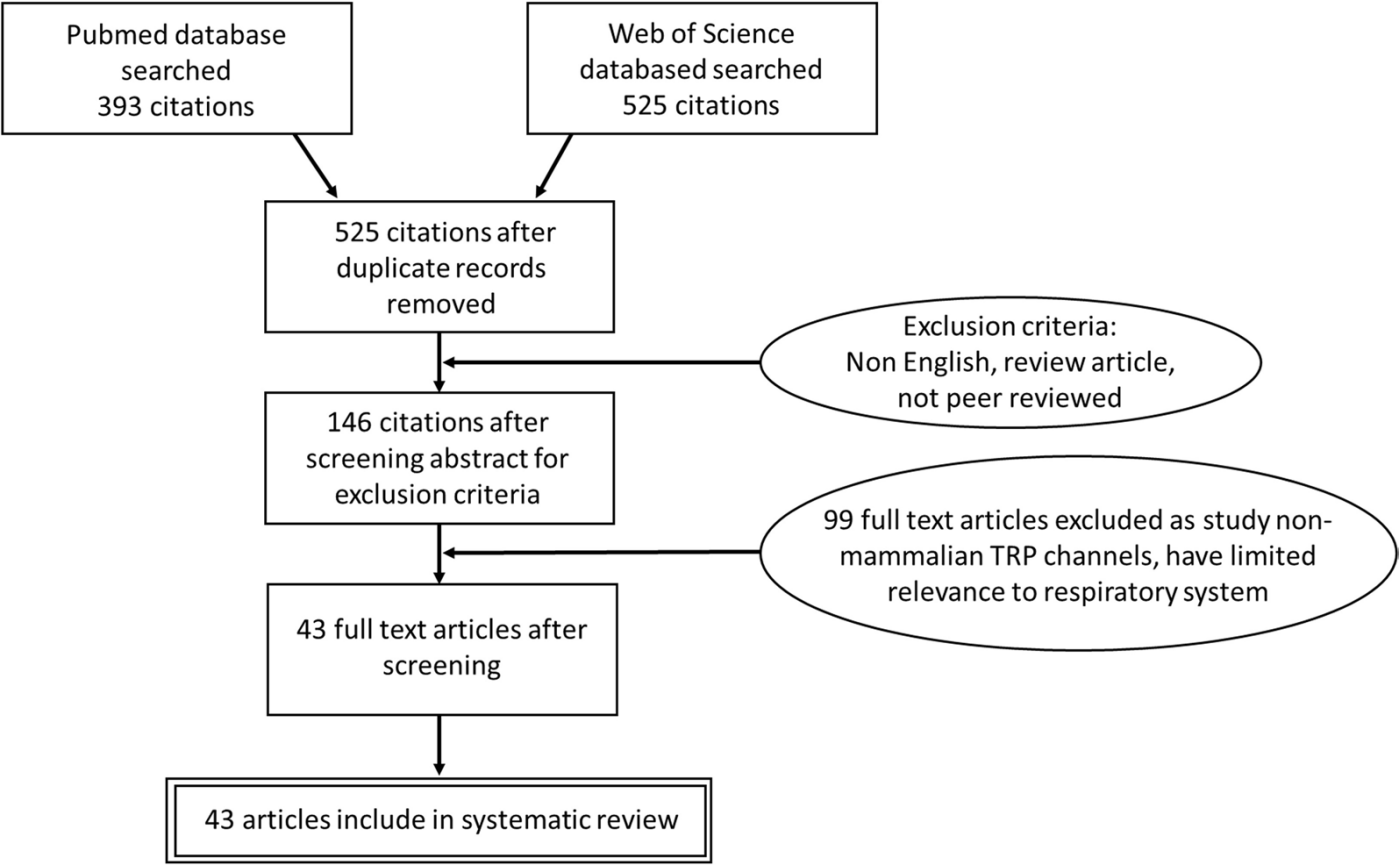
PRISMA flowchart, including exclusion criteria, utilised to screen identified citation to produce the final number of studies considered in the systematic literature search

The mechanisms involved in the action of some plant derived substances used in herbal cough and cold remedies are not well documented or explored. As such the interactions between TRP channels and the plant derived substances will be elucidated from the existing literature. To elucidate how plant derived substances, which form the major ingredients of cold remedies, may interact with TRP channels to alleviate the symptoms of the common cold, specifically cough, we searched Pubmed and Web of Science for existing studies, with no date limitations, using the principles of the PRISMA statement [69] (Fig. 3).

The following search terms were used: ‘TRP channel AND plant compound’, ‘cough AND plant compound’, ‘cough AND TRP channels AND plant compound’, ‘cough AND P2X3 AND plant compound’ and ‘P2X3 AND plant compound’ where plant compound represents menthol or camphor or eucalyptus or turpentine or thymol. Only peer reviewed, primary research articles published in English which included specified key terms were selected for inclusion in the systematic literature search (Table 2). When thymol was searched for using the same search terms as previously outlined, four articles were included, with the main observations outlined below.

**Table 2:** Publications included in the final systematic literature search

Citation	Publication type	Title	Plant derived substance and drug target
Alpizar et al*.* 2013 [99]	Original research	Bimodal effects of cinnamaldehyde and camphor on mouse TRPA1	Camphor as a TRPA1 agonist and antagonist
Andrè et al*.* 2009 [100]	Original research	Transient receptor potential ankyrin receptor 1 is a novel target for pro-tussive agents	Camphor as a TRPA1 antagonist and antitussive
Behrendt et al. 2004 [78]	Original research	Characterization of the mouse cold-menthol receptor TRPM8 and vanilloid receptor type-1 VR1 using a fluorometric imaging plate reader (FLIPR) assay	Menthol as a TRPM8 agonist
Ben-Arye et al*.* 2011 [15]	Randomised controlled trial	Treatment of Upper Respiratory Tract Infections in Primary Care: A Randomized Study Using Aromatic Herbs	Menthol, camphor and eucalyptus as an antitussive
Bödding et al*.* 2007 [81]	Original research	Characterisation of TRPM8 as a pharmacophore receptor	Menthol as a TRPM8 agonist
Buday et al*.* 2018 [121]	Randomised controlled trial	Modulation of cough response by sensory inputs from the nose—role of trigeminal TRPA1 versus TRPM8 channels	Menthol as an antitussive
Cohen et al. 2012 [126]	Randomised controlled trial	Effect of Honey on Nocturnal Cough and Sleep Quality: A Double-blind, Randomized, Placebo-Controlled Study	Eucalyptus honey as an antitussive
Ghosh et al. 2020 [109]	Original research	Essential Oils from Monarda fistulosa: Chemical Composition and Activation of Transient Receptor Potential A1 (TRPA1) Channels	Thymol as a TRPA1 agonist
Johnson et al. 2018 [122]	Observational study	Menthol Cough Drops: Cause for Concern?	Menthol as an antitussive
Karashima et al. 2007 [93]	Comparative study	Bimodal action of menthol on the transient receptor potential channel TRPA1	Menthol as a TRPA1 agonist and antagonist
Kenia et al. 2008 [20]	Randomised controlled trial	Does inhaling menthol affect nasal patency or cough?	Menthol and eucalyptus oil as an antitussive
Kumar et al. 2012 [124]	Original research	Effect of nitrogen insertion on the antitussive properties of menthol and camphor	Menthol and camphor as an antitussive
Kurohane et al. 2013 [94]	Original research	Lack of transient receptor potential melastatin 8 activation by phthalate esters that enhance contact hypersensitivity in mice	Menthol as a TRPM8 agonist
Laude et al. 1994 [125]	Original research	The antitussive effects of menthol, camphor and cineole in conscious guinea-pigs	Menthol, camphor and eucalyptus as an antitussive
Lee et al. 2008 [111]	Original research	Thymol and related alkyl phenols activate the hTRPA1 channel	Thymol as a TRPA1 agonist
Macpherson et al. 2006 [92]	Comparative study	More than cool: Promiscuous relationships of menthol and other sensory compounds	Menthol and camphor as TRP channel agonist and antagonist
Mahieu et al. 2007 [83]	Original research	TRPM8-independent menthol-induced Ca2 + release from endoplasmic reticulum and Golgi	Menthol as a TRPM8 agonist
Mälkiä et al. 2007 [161]	Original research	Bidirectional shifts of TRPM8 channel gating by temperature and chemical agents modulate the cold sensitivity of mammalian thermoreceptors	Menthol as a TRPM8 agonist
Marsakova et al. 2012 [101]	Comparative study	Pore Helix Domain Is Critical to Camphor Sensitivity of Transient Receptor Potential Vanilloid 1 Channel	Camphor as a TRPV1 agonist
McKemy et al. 2002 [84]	Original research	Identification of a cold receptor reveals a general role for TRP channels in thermosensation	Menthol as a TRP channel agonist
Mergler et al. 2013 [79]	Comparative study	Functional significance of thermosensitive transient receptor potential melastatin channel 8 (TRPM8) expression in immortalized human corneal endothelial cells	Menthol and eucalyptus as an agonist for TRPM8
Millqvist et al. 2013 [118]	Randomised controlled trial	Inhalation of menthol reduces capsaicin cough sensitivity and influences inspiratory flows in chronic cough	Menthol as an antitussive
Morice et al. 1994 [120]	Clinical trial	Effect of inhaled menthol on citric acid induced cough in normal subjects	Menthol as an antitussive
Paschke et al. 2017 [87]	Original research	Activation of the cold-receptor TRPM8 by low levels of menthol in tobacco products	Menthol as a TRPM8 agonist
Paul et al. 2010 [18]	Randomised controlled trial	Vapor Rub, Petrolatum, and No Treatment for Children With Nocturnal Cough and Cold Symptoms	Menthol, camphor and eucalyptus as an antitussive
Peier et al. 2002 [75]	Original research	A TRP channel that senses cold stimuli and menthol	Menthol as a TRPM8 agonist
Pertusa et al. 2014 [76]	Original research	Bidirectional modulation of thermal and chemical sensitivity of TRPM8 channels by the initial region of the N-terminal domain	Menthol as a TRPM8 agonist
Pertusa et al. 2018 [77]	Comparative study	Critical role of the pore domain in the cold response of TRPM8 channels identified by ortholog functional comparison	Menthol as a TRPM8 agonist
Plevkova et al. 2013 [80]	Original research	The role of trigeminal nasal TRPM8-expressing afferent neurons in the antitussive effects of menthol	Menthol as a TRPM8 agonist and antitussive
Sabnis et al. 2008 [40]	Original research	Human lung epithelial cells express a functional cold-sensing TRPM8 variant	Menthol as a TRPM8 agonist
Selescu et al. 2013 [102]	Original research	Camphor Activates and Sensitizes Transient Receptor Potential Melastatin 8 (TRPM8) to Cooling and Icilin	Camphor as a TRPM8 agonist
Takaishi et al. 2012 [106]	Original research	1,8-cineole, a TRPM8 agonist, is a novel natural antagonist of human TRPA1	Eucalyptus as agonist and antagonist for TRPM8 and TRPA1
Voets et al. 2004 [91]	Original research	The principle of temperature-dependent gating in cold- and heat-sensitive TRP channels	Menthol as a TRPM8 agonist
Wang et al. 2020 [112]	Original research	Thymol activates TRPM8-mediated Ca2 + influx for its antipruritic effects and alleviates inflammatory response in Imiquimod-induced mice	Thymol as a TRPM8 agonist
Weil et al. 2005 [82]	Comparative study	Conservation of functional and pharmacological properties in the distantly related temperature sensors TRVP1 and TRPM8	Menthol as a TRPM8 agonist
Willis et al. 2011 [85]	Original research	Menthol attenuates respiratory irritation responses to multiple cigarette smoke irritants	Menthol and eucalyptus as an agonist for TRPM8
Wise et al. 2012 [119]	Randomised controlled trial	Sweet taste and menthol increase cough reflex thresholds	Menthol as an antitussive
Xiao et al. 2008 [95]	Original research	Identification of transmembrane domain 5 as a critical molecular determinant of menthol sensitivity in mammalian TRPA1 channels	Menthol as a TRPA1 agonist
Xing et al. 2006 [90]	Original research	Chemical and cold sensitivity of two distinct populations of TRPM8-expressing somatosensory neurons	Menthol as a TRPM8 agonist
Xu et al. 2005 [46]	Original research	Camphor activates and strongly desensitizes the transient receptor potential vanilloid subtype 1 channel in a vanilloid-independent mechanism	Camphor as a TRPV1 agonist
Xu et al. 2015 [110]	Original research	Action of thymol on spontaneous excitatory transmission in adult rat spinal substantia gelatinosa neurons	Thymol as a TRPA1 agonist
Zhang et al. 2018 [117]	Original research	1,8-cineole decreases neuropathic pain probably via a mechanism mediating P2X3 receptor in the dorsal root ganglion	Use of eucalyptus in targeting pain via P2X3 pathway
Zhou et al. 2011 [86]	Comparative study	Sensitivity of bronchopulmonary receptors to cold and heat mediated by transient receptor potential cation channel subtypes in an ex vivo rat lung preparation	Menthol as a TRPM8 agonist

### Plant extract interactions with TRP channels

#### Menthol

Menthol is a cyclic terpene alcohol derived from the plants of the Mentha species, such as natural peppermint, additionally, it can be synthesised from other essential oils. Known for its distinctive flavour and fragrance, it has been widely used medicinally for over 2000 years and is widely used in other products including confectionary, toothpaste and cold medication [70–72]. Natural menthol exists in two isomer, *d*- or (+)-menthol and *l*- or (-)-menthol, with the former lacking medicinal properties [73]. Additionally, menthol has cooling and analgesic properties however, some adverse effects are linked to over exposure including irritation, skin allergies and burning sensations. Furthermore, in young children and chronic obstructive pulmonary disease (COPD) patients, over exposure has been linked to upper airway spasms, reflex apnoea and breathing difficulties [73, 74].

Menthol is a widely recognised TRPM8 agonist, as the cooling effect generated by its inhalation or topical application activates the cold sensitive channels on sensory neurons [75]. This cooling effect occurs as a result of the menthol binding to the N-terminal domain of the channel [76, 77] with (–)-menthol being a more effective agonist than (+)-menthol [78–80]. Being a voltage dependent channel, menthol binding leads to the depolarisation of the ion channel, shifting the voltage dependence of the channel to the left, nearer to the membrane potentials which are physiologically relevant for opening [81, 82]. However, it should also be noted that the C-terminal domain also plays some role in channel activation [77]. This activation results in the influx of calcium from both extracellular and intracellular sources, with the latter being via a TRPM8 independent mechanism [79, 83]. This method of activation occurs in both neuronal and non-neuronal TRPM8 expressing cells, including the bronchial epithelial cells. Activation of TRPM8 channels in the lungs via cold sensitisation may play a role in managing airway homeostasis in response to changes induced by exposure to cold air or cooling agents [40]. The cooling sensation has the benefit of inhibiting respiratory reflexes and irritation, hence the antitussive effect, but the initial activation of TRPM8 in nasal trigeminal afferent neurons may play more of a role than those situated in the bronchopulmonary vagal afferent neurons [80, 84–86]. The activation of TRPM8 in nasal trigeminal afferent neurons may go some way to explain the perceived congestion relief experienced when menthol is inhaled, creating a cooling effect and greater perception of airflow within the nose, albeit not to a measurable extent [20, 80]. The ability of menthol to inhibit respiratory irritation is not only of benefit in OTC medication for cold symptoms and as an analgesic [82], but also formed one of the major additives in cigarettes whereby, it was used to reduce the irritation caused by tobacco smoke [87]. However, it is worth noting that this practice has been banned in Europe and USA since 2020 and 2021, respectively [88, 89].

The dosage of menthol also plays some role in the sensation experienced, with low dosages causing a cooling sensation and higher dosages a burning sensation [90]. The extent of sensation experienced could reflect the number of menthol sensitive or insensitive neurons, with sensitive neurons having higher expression of TRPM8 and lower thresholds to cold stimuli [90, 91]. In addition, menthol also acts on the warm receptor TRPV3, thus activation of this receptor may explain why high concentration of menthol cause burning sensations [84, 92]. The activation of TRPA1 by menthol may explain the role it can play as an analgesic, as activation of TRPM8 alone does not cause increased skin sensitisation. Instead activation of TRPA1 by menthol can have a bimodal effect, with low doses causing pain and inflammation and high doses acting as an antagonist hence the analgesic effect [93–95], thus analgesic effects are potentially a consequence of TRPM8 activation and TRPA1 inhibition [82].

#### Camphor

Camphor is a derived from the wood of Camphor laurel and other trees of the laurel family. Native to East Asia, the distillation and purification of the wood creates an essential oil with a distinct aroma and flavour, that has long been utilised in traditional medicines [96]. Camphor relieves irritation and itch, alongside acting as an antiseptic and analgesic. It is used in topical pain relief ointments and balms, or as an inhalant to ease nasal congestion [97]. Whilst camphor has a number of benefits there are also potential risks associated with accidental ingestion or intranasal application of liquid or semi-solid camphor products, these typically include gastrointestinal symptoms, seizures and neurological changes [98].

Camphor has been less widely explored in relation to its effect on TRP channels than menthol, nevertheless camphor has been implicated as an agonist or antagonist in three different channels, namely TPRA1, TRPM8 and TRPV1. Camphor has been identified as having a bimodal effect on TRPA1, whereby, higher concentrations create an antagonistic effect and lower concentrations create an agonist response. However, these concentrations being relatively close (600 μM and 300 μM, respectively) mean that the antagonist effect may mask the agonist response [99]. Although this antagonist effect is widely documented, there is limited evidence to suggest this has any impact on cough [100]. Alongside being able to inhibit TRPA1, camphor has also been shown to activate TRPV1 via the outer pore domain of the N-terminus [101]. However, the exact mechanism by which this binding and activation of TRPV1 occurs is less clear [46]. A few potential mechanisms have been proposed including direct binding to the channel resulting in its opening or indirect opening as a response to camphor initiating a signalling pathway [46, 101]. Although camphor activates TRPV1, its efficacy is lower than other agonists, such as capsaicin, requiring concentrations in the millimolar range. Furthermore, increased temperatures also increase the activity of the channel, thus if utilised during events of inflammation or irritation, the activation of TRPV1 could be effective in creating the burning sensation, desensitisation and analgesic effect experienced when applying camphor containing balms [46]. Camphor has also been shown to activate the TRPM8 channel, at temperatures in the physiological range of the cool activated channel and with concentrations similar to those of menthol. Furthermore, camphor appears to have a bimodal effect, blocking menthol activation of TRPM8, in addition to activating the channel [102].

#### Eucalyptus oil

Eucalyptus oil, derived from the native Australia tree foliage of *Eucalyptus* species, has been utilised for hundreds of year [103]. It has a number of medicinal properties including antimicrobial, analgesic, antioxidant, anti-inflammatory and H1 receptor antagonism, as well as potential cancer therapy [104]. Over exposure can lead to dry itchy skin and burning sensations, whilst accidental ingestion can lead to gastrointestinal upset, central nervous system depression, plus cardiovascular and respiratory complications [105].

Eucalyptus oil has been linked to activation of TRP channels however, the extent of research is limited and often done in conjunction with menthol [78, 79, 84]. Eucalyptus oil is a TRPM8 agonist, activating receptors on sensory neurons, albeit to a lesser extent than on menthol [78, 79]. Eucalyptus oil is comprised of numerous chemical components, including predominately 1,8-cineole and some 1,4-cineole, which affects the manner in which it interacts with TRP channels. 1,8-cineole has been shown to activate TRPM8 whilst acting as an antagonist of TRPA1, this is potentially due to the chemical structure being similar to menthol. Conversely, 1,4-cineole activates both TRPA1 and TRPM8 however, neither have any effect on TRPV1. This bimodal effect of 1,8-cineole may indicate that eucalyptus oil could form a useful analgesic and anti-inflammatory as it does not activate TRPA1 in the same manner as menthol [106]. As with menthol and camphor, how this plays a role in the respiratory system is less clear however, inhalation of eucalyptus oil vapours gives the sensation of a clearer nose and may reflect activation of TRPM8 in the nasal passages [107].

#### Thymol

Thymol has antitussive, antibacterial and expectorant properties [108]. Thymol is often included in herbal remedies and has been shown to activate TRP channels in a similar manner to other plant derived substances utilised in these remedies. Although not extensively researched in relation to TRP channel activation, thymol has been shown to activate TRPA1 at micromolar concentrations, leading to intracellular calcium flux [109, 110]. Whilst the actual mechanism involved is not clear, thymol appears to directly activate TRPA1 and the action can be blocked by camphor. Furthermore, thymol appears to have a faster activation than other TRPA1 agonists such as cinnamaldehyde, suggesting thymol acts via a different mechanism, or binding site [111]. In addition, thymol has a bimodal effect, both activating and inhibiting TRPA1 receptor at high concentrations [111]. This activation of TRPA1 may explain the role thymol can play in pain relief [110]. Alongside TRPA1, TRPM8 has also been shown to be activated by thymol in a manner similar to menthol. Activation of TRPM8 by thymol may mean it also has an anti-inflammatory effect [112]

Whilst not included in our systematic review, cedarleaf oil has traditional use in the treatment of URTI symptoms and wounds, acting as an antiviral and antibacterial agent [113]. In addition, nutmeg oil functions as an anti-inflammatory, antiseptic, antimicrobial, analgesic and antioxidant [114, 115]. It is also worth noting that the systematic search of the literature did not produce any evidence of interactions between turpentine oil and any of the TRP channels included. However, turpentine oil has numerous beneficial medicinal properties including acting as a disinfectant, expectorant, antiseptic and antiparasitic, it is used in the treatment of bronchitis, and may aid in transdermal drug delivery [116].

Furthermore, none of the plant derived substances included in the systematic search provided evidence of interacting with TRPV4. However, eucalyptus oil or more specifically 1,8-cineole, has been shown to interact with P2X3, resulting in the downregulation of P2X3 expression on dorsal root ganglia which subsequently creates an analgesic effect [117]. Given the potential role of P2X3 in cough, specifically the TRPV4-ATP-P2X3 pathway, the use of eucalyptus oil could potentially interact with P2X3 in the airway reducing the effect of the ATP released by TRPV4 and attenuating the cough response to some extent.

### Therapeutic potential of plant derived substances

#### Antitussive effect of plant derived substances

Menthol features widely in cough research and has repeatedly been shown to have an antitussive effect. Inhaled menthol, at concentrations of approximately 1% is effective at reducing capsaicin cough sensitivity, whereby high concentrations of capsaicin are required to cause a cough response [118, 119]. When delivered repeatedly in a measured dose, via an inhaler, menthol acts as an effective antitussive, reducing cough frequency [120]. Similarly, when delivered nasally, menthol also appears to suppress airway irritation, inhibit cough and decrease sensitivity to capsaicin. However, whether this is an effect of TRPM8 activation in the nasal passages or due to the high volatility of menthol enabling it to reach the airways is not clear [121]. However, the effectiveness of menthol as an antitussive is supported by sufferers of acute cough, whereby consumption of mentholated cough drops reduces cough symptoms, with individuals increasing the number of cough drops consumed as cough severity increases [122].

Camphor also appears to have an antitussive effect, albeit studied to a much lesser extent. Nevertheless, camphor has been included in treatments for cough since the eighteenth century, with the first commercial inhaler, patented by John Mudge in 1778, utilising it as part of the mixture (with opium) inhaled by patients to treat catarrhous cough [123]. Treatment of cough with both menthol and camphor for 5 min prior to the commencement of a citric acid cough challenge caused a reduction in cough response and latency in awake guinea pigs, demonstrating the potential role of camphor in the treatment of cough [124, 125].

The effectiveness of eucalyptus oil as an antitussive is less clear, as when used alone does not cause any noticeable reduction in cough response [20, 125]. However, eucalyptus oil is used as a carrier for menthol and so features in a number of studies, whether this alters the effectiveness of menthol is not clear [20, 120]. Furthermore, the treatment of childhood nocturnal cough with eucalyptus honey showed improved sleep, reduced cough frequency and severity after consuming a measured dose of honey, 30 min prior to the onset of sleep. However, similar results were seen with citrus and labiatae honey, suggesting the honey was causing the most pronounced effect [126]. Interestingly, thymol also has some antitussive effect. Nasal application of thymol has been shown to cause a reduction in the number of coughs when challenged with capsaicin [127] and high concentrations have been shown to exhibit some antispasmodic properties on smooth muscle of the trachea [128]. Although each of these aromatic compounds have their own properties and degree of effectiveness in treating cough, when combined, in either spray or rubbing ointment form, they also appear to have a beneficial effect on this URTI symptom, reducing the severity and incidence of nocturnal cough thus improving sleep [15, 18].

#### Decongestion effect of plant derived substances

These plant derived substances also have the potential to ease nasal congestion. As with studies relating to cough, menthol features most frequently as aromatic compounds in the treatment of URTI symptoms. Inhalation of menthol activates TRPM8 receptors within the nasal mucosa, producing a cooling sensation and giving the effect of a clearer nose [129]. Similarly, orally administered menthol causes a subjective easing of nasal congestion but no marked changes in nasal airflow measurements [70]. The absence of actual change in nasal patency [20] suggests TRPM8 may not be involved either directly or indirectly in this mechanism of nasal patency [130]. Although this sensation of decongestion appears mainly subjective, this change may be a result of small reductions in ventilation, albeit only transiently, immediately after inhalation, coupled with cold receptor adaptation either locally or centrally [131]. When camphor is mixed with other aromatics including menthol, a similar effect is seen whereby, the subjective sensation of nasal decongestion is experienced but no changes to nasal airflow resistance were observed [132]. Eucalyptus oil, thyme oil and menthol, when delivered nasally has been shown to increase ciliary beat frequency which has the benefit of improving mucociliary clearance [133]. Similarly, Myrtol® a mixture of aromatic essential oils, including eucalyptus oil, which is taken orally for respiratory disorders, showed evidence of improving mucociliary clearance and increased ciliary beat frequency both in vitro and ex vivo [134]. Thymol also plays a role in increasing mucociliary clearance [128] and has been shown to have anti-inflammatory effects in allergic disorders of the respiratory system [135]. Whilst no single aromatic compound directly eases the congestion associated with a URTI, the combined use of these aromatic compounds provides a sensation of reduced congestion, which when considered alongside the increased ciliary beat frequency and improved mucociliary clearance that a number of the plant derived substances provide and the potential anti-inflammatory effect of thymol, may mean that herbal remedies may not only provide the sensation of clearer nasal passages but actually clear some of the excess mucus experienced during a URTI.

#### Sedative effect of plant derived substances

Menthol, camphor and eucalyptus oil when combined and applied topically appear to have a sedative effect which may explain the benefit of topical application of these substances prior to sleep, thus aiding in nocturnal rest and the sensation of reduced symptoms overnight [18, 136]. Both menthol and camphor have been shown to reduce spontaneous motor activity in mice who are exposed to the vapours, this is potentially due to menthol and camphor having similar chemical structures to other known sedatives. The exact mechanisms of action are unclear however, their mode of action may be a result of either interactions with olfactory nerves or the nasal mucosa when inhaled, which subsequently act on the human γ-aminobutyric acid type A (GABA_A_) neurotransmitters creating the sedative effect [137, 138]. Yomogi oil, a traditional Japanese herbal medicine, extracted from plants of the Artemisia species contains camphor and 1,8-cineole and has been shown to have a sedative effect akin to the use of lavender oil, with 1,8-cineole having the most potential for creating this effect [139]. Thymol also has a sedative effectand appears to interact with GABA_A_ receptors leading to increased function of the neurotransmitter [140, 141]. Thus, this may explain why the use of topical vapour rub ointment may help improve sleep quality during common cold infections.

#### Analgesic effect of plant derived substances

Topical application of menthol ointments is widely used to treat muscle pain as it has been shown to work by decreasing pain sensation in the skin [71, 92, 142] and altering blood flow to the underlying tissue [143], thus application can create an analgesic effect [144, 145]. Although, topical menthol ointment application is typically used for the treatment of minor muscle injuries, the analgesic effect it creates could also be beneficial in managing the muscle pain experienced during some URTIs. The use of plant derived substances as an analgesic may have potential beyond the treatment of URTI associated muscle pain. The use of throat spray containing menthol and eucalyptus has been shown to provide targeted and localised relief of the sore throat sensation often experienced during a URTI [15]. As such the use of menthol and eucalyptus in the forms of teas, sprays and lozenges may provide beneficial relief from a sore throat in the early stages of URTIs.

## Conclusions

The use of plant derived substances for their medicinal properties have a long and varied history, being utilised in not only traditional remedies for a variety of aliments but also in a number of widely available OTC treatments, most noticeably for the treatment of the symptoms of cold and flu. Although there is no clearly defined mechanism of action for many of these traditional herbal remedies, the individual plant derived substances have properties which when combined may explain how these remedies help to alleviate symptoms of URTIs. Of most interest is the interaction between the plant derived substances and the TRP channels. The cooling effect of menthol as a result of TRPM8 activation appears to have the potential to inhibit respiratory reflexes, thus reducing irritation and acting as an antitussive [80, 84–86], whilst the bimodal action leading to the inhibition of TRPA1 may have an analgesic effect [79, 93, 95], thus targeting two of the main symptoms experienced during a cold. In contrast, camphor inhibits TRPA1, yet there is little evidence to indicate whether this influences the cough reflex [100] however, the activation of TRPM8 may build on the effects of menthol, providing the sensation of easing breathing [107]. Additionally, the activation of TRPV1 by camphor, may have an analgesic effect [46] thus continuing to enhance the effect of menthol by targeting the muscle pain associated with some cold symptoms. Eucalyptus also activates TRPM8 [78, 79] and inhibits TRPA1 [106], thus further aiding in the reduction of nasal and airway irritation, the sensation of nasal clearing and the analgesic effect relieving muscle aches. Beyond the TRP channels, the downregulation of P2X3 by eucalyptus [117] may also have the potential to disrupt the TRPV4-ATP-P2X3 pathway and attenuate the cough reflex to some extent. These plants extracts also have the potential to provide a mild sedative effect [137–139] and analgesic effect [144, 145] which may help improve sleep quality, which can be disrupted by the congestion and cough experienced during a cold. Whether the action of the plant derived substances is a consequence of interaction with the TRP ‘cough’ receptors or some other mechanism that is yet to be fully elucidated remains to be seen however, there is evidence to suggest that these plant derived substances directly target a number of cold symptoms.

When taken together, the aromatic compounds appear to have the potential to interact with the TRP ‘cough’ receptors and have a beneficial effect on the treatment of cold symptoms. However, it is worth considering the complexity of this interplay between TRP receptors and the aromatic compounds which modulate them. As such, it is less clear whether the beneficial effects observed when utilising these aromatic compounds are due to direct or indirect interactions between the receptors and the aromatic compounds, or if the effects are owing to a single ingredient or the cumulative effect of all the aromatic compound. Whilst a number of questions are still pertaining to the mechanism of action of these aromatic compounds, it is clear that the plant derived substances used in traditional herbal remedies have a multifaceted action and the potential to target multiple symptoms experienced during a cold. Thus, these plant derived substances and the therapeutic vapours they release are as relevant today as they have been for the treatment of cough and cold symptoms for centuries.

## Data Availability

Not applicable.

## Abbreviations

ANKTM1: Ankyrin-like with transmembrane domains protein 1
COPD: Chronic obstructive pulmonary disease
GABA_A_: γ-Aminobutyric acid type A
hRV: Human Rhinovirus
OTC: Over-the-counter
P2R: Purinergic P2 receptors
P2X3: Purinergic receptor 3
P2XR: Purinergic P2X receptors
RSV: Respiratory syncytial virus
TRP: Transient receptor potential
TRPA1: Transient receptor potential cation channel subfamily A (Ankyrin) member 1
TRPV1: Transient receptor potential cation channel subfamily V (Vanilloid) member 1
TRPV4: Transient receptor potential cation channel subfamily V member 4
TRPM8: Transient receptor potential cation channel subfamily M (Melastatin) member 8
URTI: Upper respiratory tract infection
